# Validation of a novel and accurate ApoE4 assay for automated chemistry analyzers

**DOI:** 10.1038/s41598-020-58841-7

**Published:** 2020-02-07

**Authors:** Sergio Veiga, Andrés Rodríguez-Martín, Guillermo Garcia-Ribas, Ignacio Arribas, Miriam Menacho-Román, Miguel Calero

**Affiliations:** 1 Biocross S.L, Valladolid, Spain; 2 Department of Neurology, Ramón y Cajal University Hospital, Madrid, Spain; 3 Department of Clinical Biochemistry, Ramón y Cajal University Hospital, Madrid, Spain; 4 Institute Ramón y Cajal for Health Research (IRYCIS), Madrid, Spain; 5 Chronic Disease Programme (UFIEC), CIBERNED, and CIEN Foundation, Queen Sofia Foundation’s Alzheimer Center, Instituto de Salud Carlos III, Madrid, Spain

**Keywords:** Predictive markers, Alzheimer's disease

## Abstract

The allele ε4 of the apolipoprotein E gene (*APOE* ε4) is the major genetic risk factor for non-dominantly inherited Alzheimer’s Disease (AD). Current techniques for *APOE* ε4 carriers identification show good accuracy but have several disadvantages that limit its implementation in a clinical laboratory. These include the need for sample preprocessing, poor automation, low throughput, requirement of additional equipment, and high cost. We followed ISO 13485 guidelines to validate the *e*4*Risk test*, a new latex-enhanced immunoturbidimetric blood assay for apolipoprotein E4 (ApoE4) determination in human plasma samples. The test showed high performance in terms of lot to lot variability, precision, interferences, reagents stability, prozone, and detectability. Furthermore, diagnostic accuracy is almost equal (99%) to the gold standard, *APOE* ε4 genotyping by polymerase chain reaction (PCR). Furthermore, we demonstrated that the *e*4*Risk test* can be adapted to any clinical chemistry analyzer, including the high throughput analyzers present in most hospitals and clinical laboratories. The *e4Risk test* versatility, low cost, and easiness provides an excellent solution for *APOE* ε4 carriers identification using the same blood sample drawn for biochemical diagnostic work-up of AD patients, which can have important advantages for patient stratification in clinical trials, preventative strategies for AD, and clinical assessment of risk for brain amyloidosis.

## Introduction

Apolipoprotein E (ApoE) is a glycoprotein involved in lipid metabolism. It is encoded by the *APOE* gene, which has three different alleles (ε2, ε3, ε4) that encode for three different ApoE isoforms (ApoE2, ApoE3, and ApoE4). These isoforms only differ at the amino acids 112 and 158. Isoform E2 has cysteine at both sites, E4 has arginine at both sites, while E3, the most common form, has a cysteine at position 112 and an arginine at position 158^1,2^. These minor sequence differences have profound consequences in protein function and ApoE isoforms have been associated with the predisposition to pathological conditions such as hyperlipoproteinemia^3^, hypercholesterolemia, coronary heart disease^4^ and Alzheimer’s disease (AD). The allele ε4 of *APOE* is the major genetic risk factor and the second most important risk factor after age for non-dominantly inherited AD (by far, the most common form). *APOE* ε4 is present in approximately 20% of the global population and in 40–60% of all patients with AD^5,6^. The presence of one allele ε4 of the *APOE* gene increases the risk of AD by 3–5 fold, while the presence in homozygosis increases the risk by 8–12 fold^7^. Furthermore, *APOE* ε4 carriers have an earlier onset of the disease^8^ and they progress faster from the prodromal stage of AD (mild cognitive impairment, MCI) to AD than *APOE* ε4 non-carriers^9^. Interestingly, ApoE4 appears to be closely related with brain amyloidosis, considered as one of the earliest events in AD pathophysiology^10^. Therefore, ApoE4 determination can provide meaningful information to physicians regarding the patient risk for brain amyloidosis and AD.

Several effective techniques are described for *APOE* genotyping, including gene-based analyses such as real-time PCR^11^ and biochemical (non-genetic) methods such as isoelectric focusing-immunoblotting^12,13^, ELISA^14^ or biochip arrays^15^. However, all of these techniques pose several limitations such as time-consuming, technically complex, need additional sample processing, require the investment on specific instrumentation, lack high-throughput capacity, or lack of fully automation. These technical disadvantages hampered in part the implementation of apoE4 analysis in the clinical routine of hospitals and laboratories.

We recently described an effective ELISA-based technique that could be adapted to immunoturbidimetry^14^ in order to eliminate these technical barriers. This assay represents a fast and cost-effective assay for ApoE4 determination (*e4Risk test*), that can be very easily implemented within the clinical routine, since it uses the same sample that other routine lab tests, without any further processing, and that can be run in any chemistry analyzer, including the high-throughput clinical chemistry analyzers present in hospitals and clinical laboratories.

Here we summarize the main experiments carried out to validate the test for scale-up and commercial distribution, under a formal Design Control for *In vitro* diagnostic (IVD) medical devices following ISO13485 guidelines. We also provide one example for its adaptation to one of the leading high-throughput chemistry analyzer Architect c16000 (Abbott).

## Results

### Lot to lot variability

A total of 40 plasma samples (8 *APOE* ε4 carriers) were analyzed with the three lots of Reagent 1(R1) and Reagent 2 (R2). Identical analytical result and 100% correlation with *APOE* genotyping by PCR were obtained with the three lots, demonstrating that *the e4Risk test* can be consistently produced using different lots of key raw material and that the three lots can be used indistinctly within the *e4Risk test* Validation.

### Analytical performance

**Figure 1:**
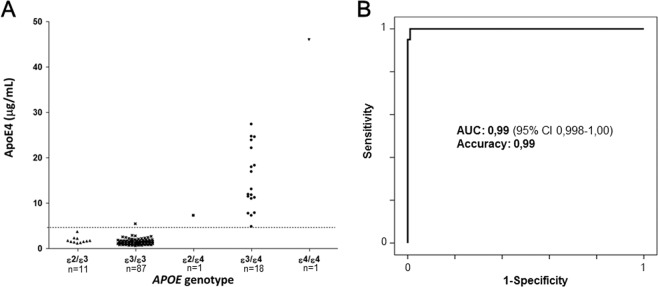
Analytical performance of the *e4Risk test* in a cohort of 118 plasma samples (20 *APOE* ε4 carriers). (**A**) ApoE4 concentration (μg/mL) against the genotype obtained by PCR. (**B**) Receiver Operator Characteristics (ROC) curve for the *APOE* ε4 carriership analysis. Analytical accuracy of the *e4Risk test* as measured as 1-misclassification rate was found to be 99%.

A total of 118 plasma samples (20 *APOE* ε4 carriers) were analyzed blinded to their *APOE* genotype. Real-time PCR showed the following genotypes (ε2/ε3, n = 11; ε2/ε4, n = 1; ε3/ε3, n = 87; ε3/ε4, n = 18; ε4/ε4, n = 1). The test showed a 100% sensitivity and 98.9% specificity. ApoE4 concentration (μg/mL) against the genotype is plotted in Fig. 1A. Receiver Operator Characteristics (ROC) curve for the *APOE* ε4 carriership analysis is shown in Fig. 1B. Analytical accuracy of the *e4Risk test* as measured as 1-misclassification rate was found to be 99% (only 1 false positive was found).

### ApoE4 signal stability

ApoE4 signal stability was analyzed in fresh and frozen plasma samples under different storage conditions and at different times after blood extraction and plasma isolation. A total of 50 plasma samples (8 *APOE* ε4 carriers) were analyzed. Fresh samples were maintained either at room temperature (RT; 22 ± 3 °C) or refrigerated (5 ± 3 °C) and analyzed immediately (t_0_), 24 hours (for RT and refrigerated conditions) or 7 days (only for refrigerated conditions) after plasma isolation. Another set of samples were immediately frozen after plasma isolation at either −20 ± 5 °C or −70 ± 10 °C and analyzed 1 month and 3 months after freezing and after 1, 2 or 3 freeze/thaw cycles. ApoE4 signal was considered stable if the same analytical result of samples (i.e. sample was classified as *APOE* ε4 carrier or *APOE* ε4 non-carrier) at t_0_ was obtained. Results showed that ApoE4 signal was stable in plasma samples at least 24 h if kept at RT and at least 7 days if kept refrigerated after plasma isolation. Similarly, plasma ApoE4 is stable in frozen plasma samples for at least 3 months and at least after 3 freeze/thaw cycles at −20 ± 5 °C or −70 ± 5 °C.

### Precision

The precision studies consisted on assaying two lots of positive controls with a theoretical concentration of 7.1 μg/mL (sample A) and 6.9 μg/mL (sample B), 2 runs per day during 20 days, every run for duplicate (a total of 80 replicates per sample). Precision data was evaluated in CV (%) of imprecision, according to guideline EP05A3E from Clinical & Laboratory Standards Institute (CLSI). Table 1 expresses the repeatability a well as the variability within laboratory, between run, within day and between days.

**Table 1:** e4Risk test precision assays, showing coefficient of variation (CV) and standard deviation (SD) of each assay for the two samples analyzed (A, theoretical concentration 7.10 μg/mL and B theoretical concentration 6.90 μg/mL).

	Mean (μg/mL)	Repeatability		Within laboratory		Between runs		Within day		Between days	
		SD	CV	SD	CV	SD	CV	SD	CV	SD	CV
A (7.10 μg/mL)	7.12	0.28	3.88	0.82	11.57	0.64	9.00	0.70	9.80	0.44	6.14
B (6.90 μg/mL)	6.32	0.20	3.21	0.67	10.64	0.34	5.31	0.39	6.21	0.55	8.64

CV is expressed in %.

Results showed that CV for these parameters did not exceed 10%, with the exception of within-laboratory variability which was slightly above (11.6% and 10.6% for samples A and B, respectively).

### Detectability (LoD and LoB)

Limit of Blank (LoB) and Limit of Detection (LoD) were evaluated according to guideline EP-17A2E from CLSI using the Classical Approach protocol with parametric data analysis previous normality test of raw data (Shapiro-Wilk and D’Agostino-Pearson tests).

Four different testing samples were prepared for LoB assays combining plasma from *APOE* ε4 non-carriers to achieve a final concentration of 0.44–0.77 µg/mL. Similarly, four different testing samples were prepared for LoD assays using the same plasmas used in LoB assays but spiked with recombinant ApoE4 (rApoE4) to achieve samples with low concentrations of ApoE4. LoD testing samples had a final concentration of 0.79–1.08 µg/mL. All samples were aliquoted and frozen at −70 °C until analysis. A total of 48 sample measurements per reagent lot were analyzed. (3 days × 4 samples × 4 replicates) for LoD and LoB determination.

LoB was found to be 0.87 μg/mL and LoD was 1.22 μg/mL.

### Interferences

Evaluation of interferences was performed based on CLSI EP7-A2. Control (without interference) and test samples (with interference factor added) were prepared from each analytical group (*APOE* ε4 carrier and *APOE* ε4 non-carrier) and both samples were analyzed within one analytical run. The following concentrations of each interference were tested: Rheumatoid factor (450 UI/mL), intralipid (3 g/L), human hemoglobin (500 mg/dL), free bilirubin (18 mg/dL) and HAMA (human anti-mouse antibodies, 40 ng/mL). Due to the impossibility to obtain *APOE* genotype from commercial HAMA preparations and the common presence of other interferents in these preparations, HAMA interference was simulated using a goat anti-mouse antibody. An interference was considered to exist if the analytical result of the test sample was changed in the presence of the interference. If any interference was found, the minimum concentration at which the interference appeared was determined.

No significant interferences of rheumatoid factor (450 UI/mL), free bilirubin (18 mg/dL) or HAMA (40 ng/mL) were observed with the established cut-off value (4.62 μg/mL). Positive interference was observed in *APOE* ε4 non-carrier plasma sample with 3 g/L of Intralipid test and 500 mg/dL of hemoglobin. These interferences were no longer observed at 2.5 g/L and 300 mg/dL, respectively.

### Prozone

Prozone was evaluated using plasma samples from *APOE* ε4 non-carriers spiked with increasing concentrations of rApoE4 (5, 10, 15, 20 and 30 times more concentrated than cut-off value (4.62 μg/mL)). Prozone effect was considered if the analytical result of a sample containing rApoE4 became negative. No prozone effect was found at any concentration, including the highest concentration tested (146 μg/mL).

### Reagents stability

Three lots of R1 and R2 were evaluated at different times after production under the following conditions: *onboard* (reagents were left opened within the analyzer at 2–8 °C and were not mixed before analysis), *shelf-life* (reagents were stored at 2–8 °C and never used/opened before analysis) and *opened* (reagents were stored at 2–8 °C, analyzed and kept closed at 2–8 °C until following analysis). Similarly, two lots of calibrator and two positive and negative controls were produced, kept at 2–8 °C and analyzed under the following conditions: Freeze-dried (vials were stored at 2–8 °C and resuspended immediately before analysis) and reconstituted (vials were reconstituted and kept at 2–8 °C reconstituted until next analysis) and evaluated at different time points thereafter.

R1 and R2 stabilities were evaluated using one aliquot of calibrator and controls as well as one plasma sample from *APOE* ε4 carrier and one plasma sample from *APOE* ε4 carrier kept at −70 °C. R1 and R2 were considered stable if the analytical result of plasma samples were unchanged, positive and negative control were under specifications and calibrator concentration remained within ±20% of assigned concentration. On the other hand, calibrator and controls were considered stable if readings remained within the initial concentration ± 20%. The test was not considered failed until two consecutive time points did not meet the criteria.

R1 and R2 showed to be stable onboard at least for 4 weeks. R1 and R2 remained stable for at least 6 months (shelf-life) and at least 8 months (opened) (stability studies still ongoing). Calibrator and controls were stable after resuspension for 1 day and 7 days, respectively. Calibrator and controls shelf-life stability is still ongoing, and so far freeze-dried calibrator and controls are stable for at least 6 months.

### Adaptability to different chemistry analyzers

The *e4Risk test* prototypes had shown prior to Validation its versatility for use in three different chemistry analyzers of different throughput capacity (Selectra Vital Junior, SpinReact, 110 tests/hour, LIDA 300 (Linear Chemicals, 300 tests/hour) and Architect c16000 (Abbott, 1600 tests/hour). Table 2 shows the optimal analytical performance of the *e4Risk test* prototype in these analyzers. Importantly, the *e4Risk test* final formulation maintained its adaptability to different chemistry analyzers such as SK-500 Biolis 50i (used during validation) and Architect c16000. Table 2 compares the analytical performance of the *e4Risk test* in both analyzers showing that the *e4Risk test* validated formulation maintained its adaptability and excellent performance among chemistry analyzers, including leading high throughput chemistry analyzers.

**Table 2:** Analytical performance of the *e4Risk test* prototype and the *e4Risk test* final formulation in 4 analyzers with different throughput capacity.

Chemistry analyzer	*e4Risk test* prototype			*e4Risk test*	
	SpinLab 100 (Spinreact)	LIDA 300 (Linear Chemicals)	Architect c16000 (Abbott)	SK-500 (Tokyo Boekis)	Architect c16000 (Abbott)
Throughput (tests/h)	110	300	1600	580	1800
N (*APOE* ε4 carriers)	114 (59)	103 (33)	103 (33)	118 (20)	80 (15)
Sensitivity (%)	100	100	100	100	100
Specificity (%)	94.8	95.9	97.3	98.9	98.5
Accuracy (%)	97.4	97.1	98.0	99.1	98.75

Accuracy is calculated as 1 minus misclassification rate.

## Discussion

We developed and validated a novel latex-enhanced immunoturbidimetric assay, the *e4Risk test*, which represents a fast, cost-effective and accurate method to identify *APOE* ε4 carriers within the clinical routine in a high-throughput and automated manner.

The *e4Risk test* is intended to be used for classifying individuals as *APOE* ε4 carrier (at-risk for AD) or *APOE* ε4 non-carrier. Identification of people at risk can be very valuable for stratification in clinical trials, prevention programs as well as providing valuable information to clinicians helping in the diagnostic process in selected cases^16,17^. The absolute concentration of ApoE4 is not informative for these intended uses and therefore, the *e4Risk test* was designed to report results qualitatively. Absolute concentrations values of ApoE4 provided by the *e4Risk test* should not be interpreted beyond its intended use. Individuals are binary classified either in *APOE* ε4 carrier or *APOE* ε4 non-carrier) depending if their apoE4 concentration is above or below the cut-off level, respectively. For example, absorbance values resulting in ApoE4 concentrations below the established cut-off value of 4.62 μg/mL are considered non-specific agglutination and will be reported as *APOE* ε4 non-carrier, despite being assigned with a positive absolute concentration of ApoE4.

We found that performance parameters regarding lot to lot variability, ApoE4 sample stability, precision, detectability, interferences, prozone, and reagents stability are good, comparable to similar routine lab tests and compatible with routine work in hospitals and clinical laboratories.

Diagnostic accuracy of the *e4Risk test* was found excellent (99%; sensitivity = 100% and specificity = 98.9%). One sample out of 118 was misclassified (false positive) in the validation set, which was also observed in the set of samples used for the adaptation to Architect c16000 (1 false positive out of 80 samples). The source of this misclassification is currently under investigation. As demonstrated during validation, high concentrations of triglycerides, cholesterol or hemoglobin, can affect the analytical results. Additionally, preliminary data from our lab suggest that the presence of anti-BSA antibodies in plasma can also interfere with the analytical results. Finally, since 90% of circulating ApoE4 is produced in the liver^18^, liver transplantation might induce some misclassification of patients if *APOE* ε4 genotype is not the same between donor and recipient. Therefore, our test should not be indicated for liver-transplanted individuals.

Higher signal was observed in *APOE* ε4 homozygous, but whether the test can differentiate by its own between heterozygous and homozygous cannot be ascertained since only 1 sample from homozygous could be recruited. Further experiments are underway in this regard. Nevertheless, the *e4Risk test* offers a sufficient level of information in order to fulfill its intended use of detecting the presence of ApoE4, independently of heterozygosis or homozygosis.

As opposed to currently available techniques for ApoE4 determination, the *e4Risk test* presents several advantages that allow its implementation within the clinical routine: First, the analysis can be performed in the same sample that other routine tests, without any preprocessing, which facilities its inclusion in routine laboratory test panels, such as the battery of tests performed in patients being evaluated for cognitive disorders (such as TSH, Vitamin B12, renal and hepatic function tests). Interestingly, these tests are not specific for AD but are used to rule out the so-called secondary dementias. The inclusion of the *e4Risk test* in this panel would provide clinicians with relevant information directly related to AD. Second, the *e4Risk test* does not require the laboratory to acquire specific instrumentation, since it can be run in the standard automated high-throughput chemistry analyzers present in most hospitals and clinical laboratories.

We believe that recent changes in scientific knowledge and AD health strategies created a demand for a fast, cost-effective and accurate method for detection of *APOE* ε4 carriers within the laboratory routine in a high-throughput and automated manner, without all the logistic problems of *APOE* genotyping by PCR.

On one hand, there is a growing literature showing that ApoE4 is directly related to extracellular amyloid beta (Aβ) accumulation (brain amyloidosis), considered one of the earliest events in AD pathophysiology^10^. ApoE4 stimulates the transcription of Amyloid Precursor Protein and Aβ secretion^19^ and also favors Aβ deposition and toxicity by several mechanisms^20,21^. All these findings obtained in animal and *in vitro* models find their correlation in humans, where it was demonstrated that the likelihood of having brain amyloidosis is significantly higher in *APOE* ε4 carriers within the entire AD continuum. Accordingly, abnormal Aβ brain accumulation occurs in more than 90% of *APOE* ε4 carriers diagnosed with AD^22^ and it was found that the probability of amyloid positivity of MCI patients is approximately twice as higher in *APOE* ε4 carriers than in *APOE* ε4 non-carriers^23^. Furthermore, even in healthy cognitively individuals, abnormal Aβ concentrations were found 10 years earlier in *APOE* ε4 carriers^24^. Therefore, ApoE4 detection provides clear information about the natural predisposition of *APOE* ε4 carriers for Aβ brain accumulation and their risk for brain amyloidosis, which is considered necessary for AD diagnosis by the latest guidelines for AD research^25^ and is has been recently proven to be a decisive information for AD diagnosis within clinical settings^26^.

On the other hand, prevention has become a major objective in the AD Health strategy. The failure during the last two decades of all investigational drugs led to the idea that treatments should be directed to very early stages of the disease^27–29^. Identification of people at risk is key for the effectiveness of preventative measures and consequently, *APOE ε4* carriers were defined as a priority population for prevention^30,31^. Accordingly, several ongoing, prevention clinical trials describe *APOE* ε4 as an inclusion factor (NCT02565511, NCT03131453, NCT02569398) and large-scale screening programs are being established to identify *APOE* ε4 carriers for AD preventive clinical trials^32^. The *e4Risk test*, due to its ease of use and low cost can be very useful as a screening test for AD clinical trials, where *APOE* ε4 carriers can be selected in a fast and inexpensive manner.

Additionally, some evidence from clinical trials and clinical registries showed that the response to several investigational and prescription drugs to treat AD symptoms is different depending on *APOE* ε4 carriership^33–39^. Therefore, ApoE4 determination would not only provide clinicians with meaningful information about patient Aβ biology and risk to AD but also can eventually help to decide the optimal medical intervention.

## Methods

### Test description and reagents composition

*The e4Risk test* is a CE-marked and commercially available immunoturbidimetry test composed of two ready-to-use reagents (R1 and R2), one calibrator, and positive and negative controls, which are used as quality controls of the assay.

R1 is provided as a solution containing an anti-apoE4 antibody in a Bis-Tris buffer, BSA, and sodium azide. R2 is a suspension containing latex particles, in a Tris buffer with sodium azide. Calibrator and controls are provided as a lyophilized powder. Calibrator consists of a matrix composed of a pool of plasmas from *APOE* ε4 non-carriers and sucrose, spiked with human recombinant ApoE4. Positive and negative controls consist of a pool of plasmas from *APOE* ε4 carriers and *APOE* ε4 non-carriers.

### Materials and reagents

The following key raw materials were used during the validation process:

Human recombinant ApoE4 (rApoE4, Peprotech; London, UK), mouse anti-apoE4 (4E4 clone, Novus Biologicals; Abingdon, UK), bovine serum albumin (BSA, Merck; Spain), human plasma (Diaserve Laboratories GmbH; Ifeldorf, Germany), latex particles (Ikerlat Polymers; Guipúzcoa, Spain), human hemoglobin, bilirubin, and intralipid were purchased from Sigma-Aldrich (Spain), rheumatoid factor (Access Biologicals, Spain), goat anti-mouse IgG (Novus Biologicals; Abingdon, UK).

#### Validation process

Three lots of R1 and R2 were produced for validation: lot A (250 mL of each reagent), lot B (500 mL), and lot C (750 mL), combining different lots of key raw material. The three lots were compared in the lot to lot variability assay as well as during reagent stability assays. The calibrator curve stability, prozone, and detectability assays were performed with two lots (B and C), while just one lot (B or C) was used for the correlation, calibrator and control stability assays, precision, interferences, and ApoE4 sample stability assays.

Two different lots of calibrator and controls were also produced for validation with the following ApoE4 concentration: Calibrators (6.44 and 6.65 μg/mL), positive controls (7.05 and 7.78 μg/mL) and negative controls (0.83 and 1.02 μg/mL). All assays were calibrated with a calibrator lot that was produced prior to validation at 7.1 μg/mL and that was aliquoted and stored at −70 °C.

#### Plasma samples and APOE Genotyping

Blood samples were centrifuged at 2280 g for 5 minutes to isolate plasma. Human plasma samples were collected from two different sources:

A set of 19 samples were acquired from Diaserve Laboratories GmbH (Iffeldorf, Germany). These samples were used for building the matrix of calibrator and controls and for the analytical performance study. Additionally, another set of samples were collected from 100 healthy volunteers (51 women and 49 men from 18 to 63 years old) within the context of the project entitled “Study for the development of a non-genetic test in blood for the identification of carriers of the ε4 allele of the *APOE* gene (*APOE* ε4)” (Code: BCR-2017-01). The study was approved by the Clinical Investigation Ethical Committee from the Santa Creu i Sant Pau Hospital (Barcelona, Spain). These samples were used in the analytical performance study as well as in the rest of the validation assays.

All samples were obtained after an informed consent form was signed by subjects. All the data were analyzed anonymously, and clinical investigations have been conducted according to the principles expressed in the Declaration of Helsinki. For logistic reasons, all plasma samples used during the *e4Risk test* validation were frozen and stored at −70 °C, with the exception of some samples used in the ApoE4 sample stability assay. Frozen samples had a maximum of 2 freeze/thaw cycles and were stored at −70 ± 10 °C for less than 1 year before analysis.

Paired samples of total blood were collected from each participant for *APOE* genotyping following the method described in Calero *et al*.^40^. *APOE* genotype obtained by Real-Time PCR was used as the reference technique for comparison of turbidimetric results.

### Gold standard

The *e4Risk test* calibrators and controls are traceable to a gold standard produced prior to validation. The gold standard consisted on a lyophilized plasma from an *APOE* ε4 carrier (ε3/ε4), whose ApoE4 concentration (13.44 μg/mL) was determined by targeted mass spectrometry (multiple reaction monitoring, MRM) following a method developed *ad hoc*, based on the method described in Rezeli *et al*.^41^.

### Sample analysis

Plasma samples used for validation were analyzed with the chemistry analyzer SK-500 Biolis 50i (Tokyo Boeki, Japan, 580 tests/hour). Shortly, the analyzer was calibrated with a two point-calibration curve, using purified water as the 0 μg/mL point and resuspended the *e4Risk test* calibrator as the 7.1 μg/mL point. Then, 5 μL of plasma were incubated with 110 μL of R1 and 110 μL of R2, gently mixed and absorbance at 546 nm was obtained immediately (A_1_). Then, the mixture was incubated at 37 °C for 5 minutes and the absorbance was read again (A_2_). ApoE4 concentration was calculated by interpolation of its (A_2_-A_1_) value in the calibration curve. Quality controls were included in each run. All samples and controls were run in duplicate, with the exception of calibrator which was run in triplicate. Results were expressed as the mean of replicates. All sample were evaluated using a cut-off of 4.62 μg/mL and were defined as *APOE* ε*4* carrier or *APOE* ε*4* non-carrier depending if its ApoE4 concentration was above or below this value, respectively. The cut-off point of 4.62 μg/mL was established based on the a priori criteria that the test should be able to detect all *APOE* ε4 carriers (sensitivity = 100%). This criteria was considered to be the most beneficial for its intended uses for identifying patients at risk of brain amyloidosis and as a screening test for clinical trials or preventive initiatives.

Sample analysis for the adaptation to the high-throughput Architect c16000 (Abbott) was identical, with the exception that sample (8 μL) and R1 (160 μL) were incubated for 5 minutes before adding R2 (160 μL), and that samples were run without replicas and analyzed at 548 nm.

### Employment or leadership

MC is one of the cofounders of Biocross SL and owns stock options from the company. ARM and SV are employees of Biocross SL. M.C. and A.R.M participate in the European patent application EP 16 794 966.8-1111, entitled METHODS FOR APOLIPOPROTEIN DETECTION which is directly related to this work and was granted by the European patent Agency on 09th January 2019.
